# Advancing the quantitative characterization of farm animal welfare

**DOI:** 10.1098/rspb.2023.0120

**Published:** 2023-03-29

**Authors:** Harriet Bartlett, Andrew Balmford, Mark A. Holmes, James L. N. Wood

**Affiliations:** ^1^ Department of Zoology, University of Cambridge, Cambridge CB2 1TN, UK; ^2^ Department of Veterinary Medicine, University of Cambridge, Cambridge CB2 1TN, UK

**Keywords:** animal welfare, life cycle assessment, pigs, livestock, agriculture

## Abstract

Animal welfare is usually excluded from life cycle assessments (LCAs) of farming systems because of limited consensus on how to measure it. Here, we constructed several LCA-compatible animal-welfare metrics and applied them to data we collected from 74 diverse breed-to-finish systems responsible for 5% of UK pig production. Some aspects of metric construction will always be subjective, such as how different aspects of welfare are aggregated, and what determines poor versus good welfare. We tested the sensitivity of individual farm rankings, and rankings of those same farms grouped by label type (memberships of quality-assurance schemes or product labelling), to a broad range of approaches to metric construction. We found farms with the same label types clustered together in rankings regardless of metric choice, and there was broad agreement across metrics on the rankings of individual farms. We found woodland and *Organic* systems typically perform better than those with no labelling and *Red tractor* labelling, and that outdoor-bred and outdoor-finished systems perform better than indoor-bred and slatted-finished systems, respectively. We conclude that if our goal is to identify relatively better and worse farming systems for animal welfare, exactly how LCA welfare metrics are constructed may be less important than commonly perceived.

## 1. Introduction

Animal welfare describes an animal's health, emotional state and behaviour [1]. In order to improve animal welfare in livestock production, we need to measure it in a way that enables valid comparison of alternative production systems [2]. Efforts to identify systems capable of reducing harmful impacts of food production typically use life cycle assessments (LCAs). LCAs are systematic techniques for quantifying a diverse range of impacts (such as greenhouse gas emissions or land use) across all stages of a product's lifestyle. LCAs are internationally standardized and have been used widely to compare the impacts of products and to identify mitigation strategies. They involve four stages: (i) definition of goal and scope of the analysis, (ii) inventory of inputs and outputs, (iii) grouping of inputs and outputs into impact categories, and (iv) sensitivity and uncertainty analysis. LCAs are mostly used to assess environmental outcomes, but are increasingly being applied to other fields (e.g. nutrition [3]), where the resulting insights help guide key decisions at policy and operational levels [4]. However, less than 1% of animal product LCAs include animal welfare [5–8], in considerable part owing to a lack of compatible metrics. This means LCAs and the decisions based on them at best involve simplistic assumptions about animal welfare, for example based on proxies unrepresentative of overall welfare rather than quantitative, animal-based welfare assessments [5,9–13], or they rely on subjective measures like stakeholder panels—which can nevertheless be useful in determining the acceptability of a system to a certain group. We suggest there is therefore a significant unmet need to develop and apply animal welfare metrics that conform to LCA principles [7].

Such metrics need to incorporate several key characteristics [5], including the quality of an animal's life assessed through a wide-ranging but tractable set of welfare elements, integrated into a single score [14–19]. Metrics should incorporate the time [20] that animals experience good welfare (welfare benefit) and bad welfare (welfare cost) conditions [17,21] because it is important to distinguish between two farms with equally poor quality of life but different quantities of life-years needed to produce a unit product: products from the system requiring twice as many life-years should be associated with twice the welfare costs. We refer to welfare as costs or benefits to parallel terminology used in LCAs and environmental economics. Costs refer to negative outcomes (in this case poor welfare) and benefits to positive outcomes (good welfare). Metrics must be relative to a functional unit (i.e. the sum of welfare costs and benefits experienced by affected animals per unit production) to enable the valid comparison of contrasting ways of meeting demand—and incorporating time allows us to do this systematically by weighting the quality of life by the time required to produce a functional unit. Metrics must allow the aggregation of welfare costs and benefits across multiple farms and production stages (e.g. for systems where breeding and finishing are on separate farms). As with LCAs, metrics must have clearly defined boundaries which identify which costs and benefits are included. These should be described explicitly and should be inclusive to allow quantification of overall welfare [22], and to avoid problem-shifting [23]. Existing methods typically lack one or more of these characteristics. For example, they may focus on quality of life and exclude the effects of time; rely on a narrow set of proximate measures of unrepresentative of overall welfare; fail to consider both beneficial and costly welfare or to address the transition from one to the other; or not test the robustness of their chosen metric using welfare data from a broad range of real-world farming systems [5,24–32].

Here our aim was to address these limitations and develop LCA-compatible animal welfare metrics. We did this by applying and extending the welfare quality (WQ) scoring system [33,34], a comprehensive and widely used method for quantifying quality of life [25]; note, however, that our approach could also be applied to other welfare scoring systems. Importantly, by itself WQ does not incorporate how long animals must experience a level of quality of life (quantity of life-years), is not relative to a functional unit, does not provide an overall quantitative score and lacks consensus on how to determine the transition from welfare cost to benefit. In this study, we transformed WQ into several welfare-cost metrics that address these shortfalls and applied them to data from 74 UK pig systems (approx. 5% of the UK's pigs). We refer to metrics as ‘welfare-cost’ as they are constructed so that higher scores indicate poorer outcomes, in line with LCAs. However welfare-cost metrics can have negative scores, indicating systems that have net welfare benefits.

The construction of animal-welfare metrics will always be, at least to some extent, subjective because perceptions of animal welfare vary [35]. There is disagreement on the relative importance of different components of animal welfare [36] (e.g. good health versus appropriate behaviour) and on what is considered good versus poor welfare [37]. The purpose of this study is not to identify correct or accurate metrics, but (i) to develop an approach to welfare measurement which is in principle compatible with LCA approaches to assessing other impacts, and (ii) to test the sensitivity of assessments of which farming systems are better or worse for welfare to different ways of formulating welfare metrics. The metrics we explore have been developed to reflect a broad range of attitudes to welfare, including metrics that assume that all observed welfare scores are poor (costly), or good (beneficial), as well as intermediates [38,39]. We investigated the robustness to variation in the metric used of the welfare rankings of both individual breed-to-finish systems and groups of systems categorized by farm label types (taken here as memberships of quality-assurance schemes or other product labelling).

## 2. Methods

### (a) Farms

We contacted 150 pig producers in the UK by email or phone and 44 participated in the study. These participants provided data on 74 breed-to-finish systems with highly varied characteristics (table 1). We categorized systems by label type and husbandry type. Label type is defined here as membership of a farm assurance scheme, such as *Red tractor, RSPCA assured* or *Organic*; or non-assurance scheme labels such as free range, woodland; or ‘none’, those farms with no assurance or labelling*.* The *Red tractor* scheme builds on UK minimum standards. *RSPCA assured* is welfare focused, with restrictive farrowing crates not permitted, and additional enrichment requirements. *Organic* standards exceed these, require outdoor access and have strict regulations on mutilations. Free range, while not a formal assurance, typically refers to fully outdoor systems, and woodland to those with at least partial tree cover. Categorizing farms is challenging as often categories are not clearcut or mutually exclusive—farms can have several label types or none. To ensure independence of data points, where our sample systems belonged to two or more labels, we assigned them to their most demanding label (see the electronic supplementary material, figure S1 for a Venn diagram showing the overlapping label types for our 74 data points). Husbandry type is split into breeding (indoor, hybrid indoor–outdoor and outdoor) and finishing (slatted, straw yard and outdoor). Several participating producers had multiple farms, which span breeding to finishing. Breeding farms produce piglets which remain on the breeding farm until weaning. At weaning, piglets move to the fattening stage, which can take the form of either two stages (rearing and finishing farms) or a single stage (fattening farm). Each of our data points had a unique finishing or fattening farm, but some shared breeding and/or rearing farms which means our data points are not entirely independent of one another. We address this in our analyses (see Statistical analyses below).

**Table 1.** Description of the 74 breed-to-finish pig systems studied. (The label categories are approximately ordered by the degree of welfare standards required by each, with more demanding categories exceeding the standards of lower categories. From least to most demanding the categories are no assurance or labelling (none), *Red tractor* (including *Quality Meat Scotland; QMS*), *RSPCA assured*, free range, woodland and *Organic*. If systems met the requirements for multiple labels, they were included in the most demanding label type, for example: free-range systems that are also *RSPCA assured* were included in the free-range category. Any relevant label standards or guidelines can be found in the citations in the first column. The UK pigs by label type column show the percentage of the total slaughtered fattening pigs in the UK in 2021 [40] with each label type [41,42]. These sum to more than 100% as farms often have multiple label types. The pigs in this study column show the annual slaughtered fattening pigs from our 74 systems, summed by label type and rounded to the nearest 1000, and our estimate of the percentage of all slaughtered pigs belonging to that label type which they represent. In total, our study covers 5% of UK slaughtered fattening pigs.)

label type	breeding husbandry type	rearing and finishing husbandry type	number of breed-to-finish systems	UK pigs by label type (%)	pigs in this study (% of UK total pigs)
none [43]	typically indoors. Farrowing crates are permitted	typically indoors. Fully slatted floors are permitted	4	5	38 000 (7%)
*Red tractor* [44] including *QMS* [45]	typically indoors. Farrowing crates are permitted	typically indoors. Fully slatted floors are permitted	31	95	479 000 (5%)
*RSPCA assured* [46]	farrowing can be indoors, but sows must be allowed to turn around at all times	pigs must have access to unperforated floors and sufficient bedding	12 (of which 10 are also *Red tractor*)	unknown	222 000 (unknown)
free range	always outdoors		18 (of which 15 are also *Red tractor* and *RSPCA assured*)	2.5	165 000 (60%)
woodland	pigs are kept at least with partial tree cover, but farms could also include some indoor housing		3 (of which 2 are also free range)	unknown	13 000 (unknown)
*Organic* [47,48]	always outdoors		6 (of which 5 are also *Red tractor, RSPCA assured* and all 6 are free range)	0.6	31 000 (47%)

We visited each system between September 2018 and December 2020. We used a questionnaire to collect information on the quantity of life-years needed to produce the functional unit of 1 kg of deadweight (DW) averaged over the most recent year of available data, which included a minimum of one complete breeding or fattening cycle for breeding or fattening farms, respectively. Breed-to-finish systems produce DW from fattening pigs and cull sows. We equated these using economic allocation informed by mean prices from the Agriculture and Horticulture Development Board [49] and a large UK pig processor averaged over the study period. H.B. (who is WQ certified) undertook WQ assessments for sows and piglets, and separately for fattening pigs [34]. Our system boundaries included the welfare of breeding sows through gestation and lactation and fattening pigs from birth to finish. We excluded welfare at transport, slaughter, of breeding boars, and upstream welfare associated with the production of inputs such as animal-based feed or gilts. The contribution of these elements to overall welfare metric scores is likely to be small owing to the relatively small number of life-years associated with these per functional unit. Our analyses in effect assumed that these neglected elements of welfare were not negatively related to our WQ and metric scores.

### (b) Overall welfare quality score

WQ assessments are a method of quantifying quality of life at the farm level. WQ is made up of four principles, which are each made up of several criteria, which in turn are each made up of several measures [25]. These measures involve mostly animal-based assessments carried out on samples of animals and their environments. The four principles have scores ranging from 0 to 100, with 100 indicating the highest welfare. WQ scores have an indifference threshold of five points, which means scores are only considered to be biologically different if this threshold is exceeded [34]. WQ assessments are criticized for their snapshot approach. However, many WQ measures are designed to be indicative of long-term welfare; more broadly we expect that our relatively large sample of farms means that our data are reasonably representative of animal welfare over the long run.

There is currently no clear consensus on how different aspects of welfare (in this case WQ principle scores) should be combined into an overall score [2,37]. We therefore chose three alternatives to reflect this uncertainty (*w* in equation (2.1); standard weighting: $w_{1}=0.35$, $w_{2}=0.25$, $w_{3}=0.25$ and $w_{4}=0.15$; equal weighting: $w_{1}=0.25$, $w_{2}=0.25$, $w_{3}=0.25$ and $w_{4}=0.25$ and extreme weighting: $w_{1}=0.50$, $w_{2}=0.20$, $w_{3}=0.20$ and $w_{4}=0.10$). There is evidence that health is perceived to be the most important aspect of welfare [39,50], which is reflected in our standard weighting. We also explored alternatives: equal weighting, which removes the emphasis on health and treats all principles equally, and extreme weighting, which increases the emphasis on health. There is also no standardized way of combining WQ scores across multiple production stages. Therefore, for each farm, we derived an overall WQ score incorporating principle scores from the sows and piglets assessment and the fattening pigs assessment for each breed-to-finish system. We did this by weighting WQ scores by the respective proportion of life-years required by each production stage to produce a finished pig sent to slaughter:

2.1 $$\mathrm{overall}\;\mathrm{WQ}\;\;\mathrm{score}=\;\overset{i=4}{\underset{i=1}{\sum }}\{\begin{array}{c} \frac{\left(p_{i\mathrm{SP}}w_{i\mathrm{SP}}t_{\mathrm{SP}}+\;p_{i\mathrm{FP}}w_{i\mathrm{FP}}t_{\mathrm{FP}}\right)}{\left(t_{\mathrm{SP}}+t_{\mathrm{FP}}\right)} \end{array},$$

where *i* = WQ principles of 1. good health, 2. good feeding, 3. appropriate behaviour and 4. good housing; *p* = WQ principle score; *w* = WQ principle weighting; *t* = proportion of life-years needed to produce a finished pig sent to slaughter for each production stage: SP = sows and piglets; FP = fattening pigs.

*t* was calculating using data obtained through the questionnaire on productivity and animal numbers which were averaged over the most recent year of available data.

### (c) Welfare-cost metrics

Here we present several alternative ways to convert WQ scores into welfare-cost metrics which incorporate *y*, the number of life-years required to produce 1 kg of DW. In contrast with WQ scores, higher welfare-cost metrics scores are indicative of poorer welfare:

2.2 $$\mathrm{welfare}\;\;\mathrm{cost}=\;\overset{i=4}{\underset{i=1}{\sum }}\{\begin{array}{c} (100-p_{i\mathrm{SP}})w_{i}y_{\mathrm{SP}}+(100-p_{i\mathrm{FP}})w_{i}y_{\mathrm{FP}},\;p<T\\ -p_{i\mathrm{SP}}w_{i}y_{\mathrm{SP}}+-p_{i\mathrm{FP}}w_{i}y_{\mathrm{FP}},\;p\geq T \end{array},$$

where *y* was calculated using data obtained through the questionnaire on productivity, animal numbers and DW output (or liveweight and dressing percentages) which were averaged over the most recent year of available data. The number of life-years was calculated separately for sows and piglets and for fattening pigs, and reflects the number of life-years required to produce 1 kg of DW at slaughter rather than growth rates in these systems. An animal's concept of the future focuses on immediate threats or opportunities and they are unlikely to consider long-term life expectancy [51,52], so we disregard the effects of premature death. Fattening animals that died or were culled on farm, and so did not enter the food chain, were assumed to have lived the average age of those that went to slaughter as reliable data on age at death were unavailable. The total number of life-years needed to produce 1 kg of DW was insensitive to this assumption (see the electronic supplementary material, figure S2 for a sensitivity analysis). *T* = the principle score at which a welfare cost transitions to being a negative cost—which we refer to as a welfare benefit. There has been much debate around which criteria should determine *T* and no consensus has been met [17,53–56]. Here we attempted to progress understanding by presenting 10 alternatives and testing how far different approaches alter the outcomes. Most WQ measures focus on the presence or absence of indicators with negative impacts on welfare like lameness or wounds. This means generally anything but a perfect score indicates the presence of an indicator with negative implications for animal welfare. This was reflected in our first metric, where all but perfect WQ principle scores were treated as a cost (*T* = 100); this approach aligns with some perceptions of the quality of life of farmed animals [38,57]. However, the standards considered to be acceptable vary considerably [35,38,50,58,59], so we also included metrics with transitions at a range of lower principle scores (*T* = 90, 80, 70, 60, 50 and 40). We explored two further approaches for distinguishing systems which are costly versus beneficial to animal welfare. The WQ framework classifies farms with all principle scores greater than or equal to 55 and two greater than or equal to 80 as ‘excellent’, and farms with all principle scores greater than or equal to 20 and two greater than or equal to 55 as ‘enhanced’. We therefore used these two classifications as transitions (*T* = excellent, enhanced), with all principle scores treated as *P* ≥ *T* in equation (2.2) if the sows and piglets and/or fattening pigs scores met the relevant classification and *P < T* if not. Last, given calls for welfare assessments to shift from focusing on negative indicators to also include indicators of positive welfare, which are typically behavioural [60], our final metric treated the WQ appropriate behaviour principle scores as a benefit (and so *P* ≥ *T* in equation (2.2)) and scores for the other three WQ principles as a cost.

### (d) Statistical analyses

There were insufficient data to statistically remove the effects of shared breeding or rearing farms, so where statistics are reported this is for a subset of our data (*n* = 43), with one datapoint randomly selected from those that shared breeding and/or rearing farms. We used Kruskal–Wallis tests with Dunn's *post hoc* analysis using the Holm method to control for multiple comparisons to identify significant differences among label and husbandry types. We used Spearman rank correlations to test the sensitivity of system rankings to the use of different approaches to metric construction. These analyses were carried out in RStudio 4.1.1 [61] using ‘stats’, ‘FSA’ [62] and ‘tidyr’ [63], and summarized and visualized using the packages ‘rcompanion’ [64], ‘ggthemes’ [65] and ‘ggplot2’ [66].

## 3. Results

### (a) Overall welfare quality scores

**Figure 1.**
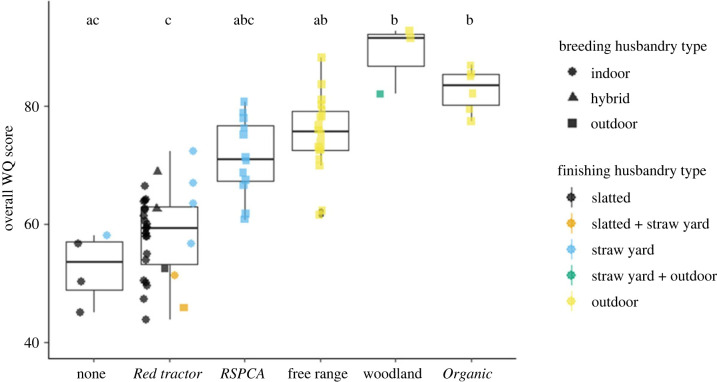
Overall WQ scores with WQ principle scores from sows and piglets and from fattening pigs combined into a single score using equation (2.1)—where each WQ score was weighted by the proportion of life-years required to produce 1 kg of DW using standard WQ principle weighting. Upper and lower whiskers extend to 1.5 times the interquartile range from upper and lower hinges, respectively. The middle horizontal bar is the median. The shapes and colours of scattered points show the husbandry type of breeding and finishing farms, respectively. Letters above boxplots show the results from Dunn's *post hoc* tests which used Holm's method to control for multiple comparisons. Different letters indicate significant differences between median values (*p* < 0.05; see the electronic supplementary material, table S1 for *p*-values). All statistically significant differences exceeded the WQ indifference threshold of 5 [34] which means they are also judged to be biologically different.

Overall WQ scores varied both within and between label types (figure 1), with significantly different medians across labels (Kruskal–Wallis *χ*^2^_5_ = 32.6, *p* < 0.01). Overall WQ scores were calculated by combining WQ principle scores from sows and piglets and from fattening pigs into an overall score by weighting them by the proportion of life-years required by each system to produce a finished pig sent to slaughter using standard WQ principle weighting (see equation (2.1)). *Post hoc* Dunn's analyses revealed that woodland and *Organic* overall WQ scores were significantly higher than those for *Red tractor* and ‘none’, free range scores were also significantly higher than *Red tractor* (see letters at the top of figure 1 and the electronic supplementary material, table S1 for *p*-values). There were also significant differences in overall WQ scores by both breeding and finishing husbandry types (Kruskal–Wallis *χ*^2^_2_ = 29.8, and *χ*^2^_4_ = 30.7, both *p* < 0.01), and *post hoc* Dunn's analyses found that outdoor-bred systems had significantly higher scores than indoor-bred, and outdoor-finished than slatted (see the electronic supplementary material, tables S2 and S3, and figure S3).

### (b) The quantity of life-years needed to produce a kilogram of deadweight

**Figure 2.**
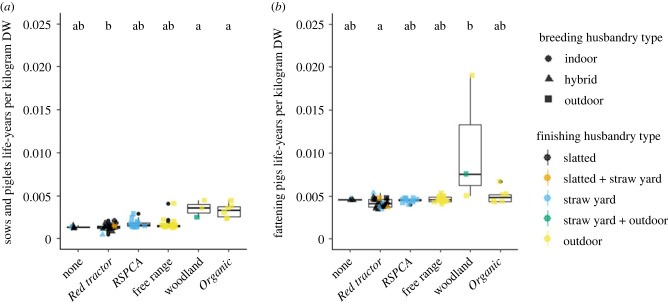
Life-years required to produce 1 kg of DW; (*a*) sows and piglets until weaning, (*b*) fattening (wean-to-finish) pigs. Upper and lower hinges correspond to the first and third quartiles. Upper and lower whiskers extend to 1.5 times the interquartile range from upper and lower hinges, respectively. The middle horizontal bar is the median. The shapes and colours of scattered points show the husbandry type of breeding and finishing systems, respectively. Letters above boxplots show the results from Dunn's *post hoc* tests with different letters indicating significant differences between median values (*p* < 0.05; see the electronic supplementary material, table S1 for *p*-values).

Label types also varied significantly in the number of life-years required to produce 1 kg of DW for sows and piglets, and fattening pigs (figure 2*a,b*; Kruskal–Wallis *χ*_5_^2^ = 24.4 and *χ*_5_^2^ = 16.0 respectively, *p* < 0.01). The number of life-years is the number of breeding sow and pre-weaning piglet life-years (figure 2*a*), and fattening pig life-years (figure 2*b*) needed to produce 1 kg of DW of a pig at slaughter, rather than a measure of growth rates. *Post hoc* Dunn's analyses revealed that woodland and *Organic* systems required more sow and piglet life-years per kilogram of DW than *Red tractor*, and woodland required more fattening pig life-years per kilogram of DW than *Red tractor* (see the electronic supplementary material, table S1 for *p*-values)*.* There were also significant differences in the number of life-years by both breeding and finishing husbandry types (Kruskal–Wallis *χ*_2_^2^ = 22.5, and *χ*_4_^2^ = 14.1, both *p* < 0.01; see the electronic supplementary material, tables S2 and S3 for *p*-values, and figure S4 for a visualization of this). *Post hoc* Dunn's analyses found that outdoor-bred systems involved significantly higher numbers of sow and piglet life-years per kilogram DW than indoor-bred, and outdoor-finished systems had more fattening pigs life-years per kilogram DW than those with slatted finishing. Systems with higher overall WQ scores required more total life-years to produce 1 kg of DW (Pearson rank *r*_p_ = 0.57, d.f. = 41, *p* < 0.01). How this relationship is viewed—as positive or a negative—depends on whether quality of life is deemed sufficiently high that more time experiencing it is beneficial to welfare.

### (c) A welfare-cost metric

**Figure 3.**
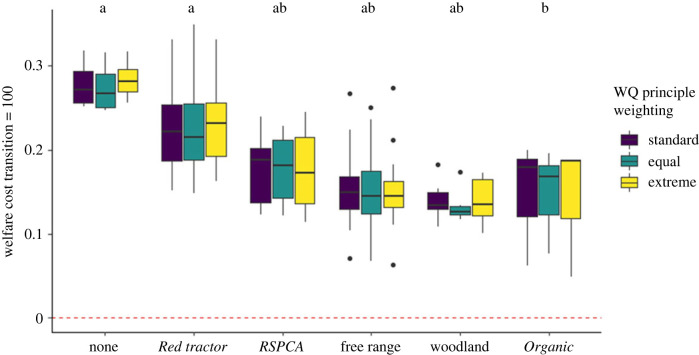
Welfare costs using a metric at which the transition from welfare cost to benefit (*T*) is set at 100. In contrast with figure 1, here higher values are indicative of poorer welfare. Upper and lower hinges correspond to the first and third quartiles. Upper and lower whiskers extend to 1.5 times the interquartile range from upper and lower hinges, respectively. The middle horizonal bar is the median. Letters above boxplots show the results from Dunn's *post hoc* tests, with different letters showing significantly different medians (see the electronic supplementary material, table S1 for *p*-values). We used Holm's method to control for multiple comparisons. The three WQ principle weighting methods (shown by the different colours) were associated with the same pairwise differences. The red dotted line is set at 0—with scores above the line being costs, and below being negative costs—i.e. benefits.

Now we turn to welfare-cost metrics, which combine WQ scores and the quantity of life-years needed to produce 1 kg of DW (see equation (2.2)). Figure 3 shows the welfare costs of a metric with *T* in equation (2.2) set at 100, so anything less than the highest possible quality of life (WQ principle scores of 100/100) resulted in life-years being treated as costly. Different possible approaches to weighting each WQ principle (*w* in equation (2.2))—which describe how each one is incorporated into an overall score—are shown by the different colours. Label types had significantly different welfare costs (Kruskal–Wallis on standard WQ principle weighting: *χ*_5_^2^ = 22.4; equal weighting: *χ*^2^_5_= 20.1; extreme weighting: *χ*^2^_5_= 24.3, *p* < 0.01), with *Organic* welfare costs significantly lower than *Red tractor* and ‘none’–those systems with no label. There were also significant differences in the welfare costs by both breeding and finishing husbandry types (Kruskal–Wallis *χ*^2^_2_ = 18.7, and *χ*_4_^2^ = 29.1, both *p* < 0.01). *Post hoc* Dunn's analyses found that outdoor-bred systems had significantly lower costs than indoor-bred, and outdoor-finished systems than those with slatted finishing (see the electronic supplementary material, tables S3 and 4 for *p*-values, and figure S5 for a visualization of this). The cost scores for different label and husbandry types were insensitive to different WQ principle weightings (Kruskal–Wallis test *p* = 0.53). This choice of transition deemed all systems studied to impose a welfare cost, which may reflect some people's attitudes to welfare [38]. We now turn to nine additional metrics which used different criteria to determine the transition from welfare cost to benefit.

### (d) Welfare-cost metrics with different transitions

**Figure 4.**
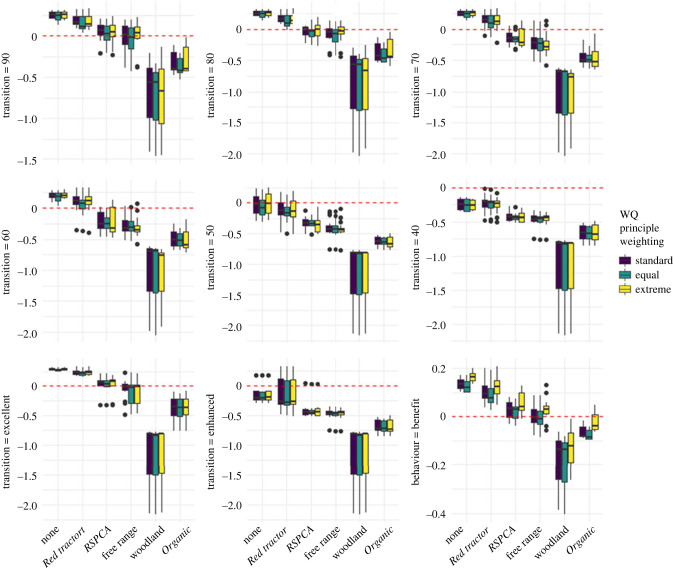
Welfare costs according to several metrics with varying criteria determining the transition from welfare cost to welfare benefit (*T* in equation (2.2)). Upper and lower whiskers extend to 1.5 times the interquartile range. The middle horizonal bar is the median. The red dotted line is set at 0—with scores above the line being costs, and below being negative costs—i.e. benefits. Pairwise significant differences from a Dunn's *post hoc* analysis can be found in the electronic supplementary material, table S1.

**Figure 5.**
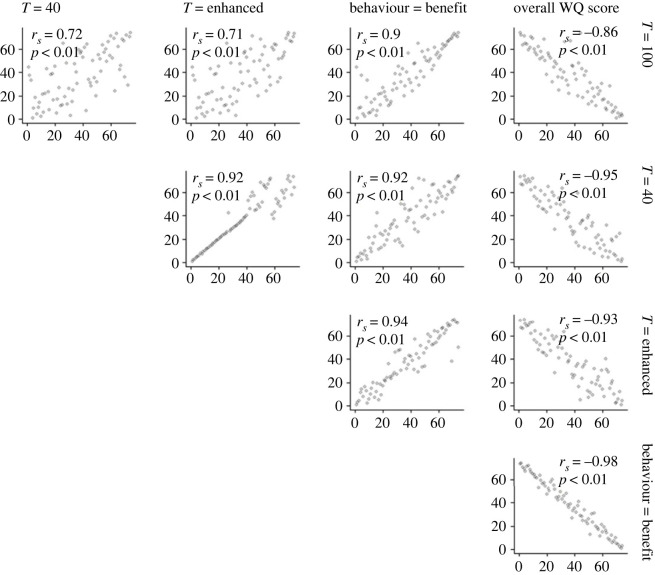
Rankings of breed-to-finish systems from low to high costs or scores according to welfare-cost metrics and overall WQ scores. *r*_s_ and *p*-values are from Spearman rank correlations based on welfare costs or overall WQ scores not the rank data show in plots. High overall WQ scores are indicative of high welfare, whereas the opposite is true for welfare-cost metrics—hence the negative relationships shown in the column furthest on the right. See the electronic supplementary material, figure S8 for the same analysis of all welfare-cost metrics, and figures S9 and S10 for alternative WQ principle weightings.

Next we explored the effects of changing the transition criteria at which WQ scores are viewed as costs or benefits to animal welfare (*T* in equation (2.2)). Unsurprisingly, changing *T* altered our overall view of how far pig production imposed welfare costs. Incrementally lowering the transitions for each principle score from 100 (figure 3) to 40 resulted in progressively more farms being rated as having negative welfare costs (i.e. benefiting welfare—figure 4, top two rows). Treating as beneficial those principle scores that triggered a farm's WQ score to be deemed excellent or enhanced had a similar effect, as did treating appropriate behaviour principle scores as beneficial (figure 5, bottom row). Importantly, however, the relative ranking of our label categories remained very largely unchanged by these different transitions. Across all the panels in figure 4, woodland systems had the highest welfare (lowest welfare costs) followed by *Organic* systems, then free range or *RSPCA assured*, then *Red tractor* and lastly ‘none’ (those systems with no assurance or labelling). Overall differences among label types in all cases remained significant (see results from Kruskal–Wallis and Dunn's *post hoc* tests in the electronic supplementary material, table S1). Most metrics resulted in woodland and *Organic* costs being significantly lower than ‘none’ and *Red tractor*, and free range lower than *Red tractor*. Woodland scores appear to consistently perform better than all other labels, but our small sample size (*n* = 3) may be limiting our ability to identify further significant pairwise differences. There were also significant differences in welfare costs by both breeding and finishing husbandry types (see the electronic supplementary material, figures S6 and S7 for plots by husbandry type, and tables S2 and 34 for results of Kruskal–Wallis analyses). Across all metrics, outdoor-bred systems had significantly lower costs than indoor-bred, and outdoor-finished systems had lower costs than those with slatted finishing. Again, these findings were not significantly different across three ways of weighting WQ principles into a single score (Kruskal–Wallis test *p* = 0.23).

### (e) The effect of metric transition on farm rankings

To check whether this similarity in relative outcomes held not just at label type rankings, but also at the level of individual breed-to-finish systems, we tested how far the system rankings correlated across different welfare-cost metrics. We found that system rankings were strongly and significantly correlated regardless of the choice of metric (see figure 5 which shows correlations for a subset of metrics using standard WQ principle weighting; see the electronic supplementary material, figure S8 for the same analysis for all metrics, and figures S9 and S10 for equal and extreme WQ principle weightings). In addition, system rankings of different welfare-cost metrics were highly and significantly correlated with overall WQ scores (see column furthest on the right in figure 5).

## 4. Discussion

LCAs typically ignore animal welfare because of a lack of compatible metrics. To include animal welfare in LCAs, we need metrics that systematically aggregate welfare consequences associated with the production of a functional unit, but there is no consensus on how this should be done. There is debate around how to combine different aspects of animal welfare (such as health versus behaviour) into a single quantitative score and what criteria should determine good versus poor welfare—which are also barriers to creating these metrics. Here we constructed several LCA-compatible metrics that take a range of approaches to these sources of contention, and we explored their consequences for 74 varied real-world farming systems. We found unsurprisingly that altering our arbitrary designation of the point of transition from a welfare cost to benefit altered how far systems, label and husbandry types imposed welfare costs. However, and, we believe, more importantly, there was broad agreement across different welfare-cost metrics in which systems, label and husbandry types are relatively better or worse: rankings were largely insensitive to the criteria used to determine the transition from costly to beneficial welfare, and the choice of how to weight WQ principles. We found, according to most animal-welfare metrics, that woodland and *Organic* farms score significantly better than those with no certification or labelling (none) and *Red tractor* farms. We also found that outdoor-bred and outdoor-finished systems perform significantly better than indoor-bred and slatted-finished systems, respectively. Our findings may be limited by sample size (e.g. to the best of our knowledge there are only three commercial woodland farms in the UK). However, this is, to our knowledge, the first study to use LCA-compatible welfare metrics built on comprehensive animal-based welfare assessments to compare the overall welfare of a diverse and large sample of systems. This study builds the foundation for future work in two ways. First, it shows that LCA principles can be applied to animal welfare quantification to enable systematic comparisons. Second, this study provides a method for including animal welfare in LCAs—it no longer needs to be assumed or ignored.

Our study was nonetheless limited by the methods used to quantify quality of life [67]. WQ protocols are used widely, but have been criticized over the choice of measures included, their focus on group-level welfare rather than on individuals, how they are combined into an overall score, the level of compensation between different aspects of animal welfare, and for their snapshot approach to welfare quantification ([1,15,45,68,69]; we expect that the effects of the latter are in part moderated by our relatively large sample of farms). There are also concerns that WQ assessments underestimate the quality of life of free-range production. While this may be the case, it would probably not affect which systems and label types are deemed relatively better or worse as increasingly free-range systems already consistently outrank indoor systems. WQ's limitations are partly because any method, to be widely adopted, needs to be accessible and efficient [2]. We suggest that our framework could be readily adapted to other welfare quantification methods now and as they develop, for example as data collection becomes more automated [70,71] and as WQ protocols improve, for example to: account for multiple welfare problems in individuals; include thresholds for unacceptable scores of individual measures [37]; and reduce unacceptable compensation in calculations of scores [18]. Our study did not include welfare assessments up or downstream of the breed-to-finish system (such as welfare costs of producing animal-based feeds or, of slaughter); future studies could include these, but they are likely to have small effects on the results owing to the relatively small contribution they make to the quantity of life-years required to produce a unit product. Our study also did not examine the acceptability of these systems to consumers and experts. Future analyses should combine empirical and systematic evaluations of animal welfare using metrics like those presented here but also consider stakeholder opinions on acceptability to eliminate any options which are societally unacceptable and help identify the most appropriate metrics.

When developing a metric that combines quality of life and quantity of life-years per unit production, there is an inescapable challenge of how to treat those life-years—whether they should be viewed as positive or negative and, if some combination of the two, what determines the transition between them. Any judgement about where the transition between ‘good’ and ‘bad’ or 'acceptable’ and ‘unacceptable’ welfare lies will always be subjective or based on assumptions [56]. However, we discovered that if the aim of the exercise is largely to identify better or poorer performing systems or categories of farms, the choice of transition is less important than perhaps assumed, as the ranking of systems, husbandry and label types was consistent regardless choice.

The quantity of life-years needed to produce a unit production raises other important considerations. It may predict other impacts like greenhouse gas and land-use footprints. Systems that require more life-years to produce a unit production are likely to have lower productivity and poorer feed conversion ratios and hence greater environmental externalities [72–76]. As such, the relationship between animal welfare and other key externalities is often assumed to be a trade-off [5,10–12,57,75,77,78]. This assumption has not been systematically tested across contrasting systems, in part owing to a lack of animal-welfare metrics. We suggest that we now examine the associations between welfare and environmental externalities empirically and use the results to support informed decisions about optimal systems, which outcomes to compromise and to what extent. Animal welfare can and should be considered alongside externalities such as greenhouse gas or land footprints in LCAs [79]. It is important to establish where there are trade-offs among externalities and to identify which systems best address a broad suite of societally important outcomes.

## 5. Conclusion

The lack of consensus on how best to quantify animal welfare has resulted in it being excluded from LCAs aimed at improving farming systems. The choice of animal welfare metric (and its components) is, of course, conceptually important. However, our findings suggest that conclusions around which systems and approaches perform best are quite insensitive to how welfare metrics are calculated. We should continue to advance animal welfare quantification, but our results indicate that we should not let a lack of consensus on relatively inconsequential details of how best to assess it result in animal welfare being excluded from LCAs comparing system performance. We show that it is possible to measure animal welfare empirically and transparently, and we propose that animal welfare should be explicitly included in LCAs using metrics which, like those presented here, combine both quality and quantity of life-years needed for a unit of production. This will enable exploration of trade-offs and synergies among animal welfare and an array of other outcomes of societal concern and help to identify which systems perform best across them.

## Data Availability

Data can be found in the electronic supplementary material [80].
